# Spatial heterogeneity and factors influencing stunting and severe stunting among under-5 children in Ethiopia: spatial and multilevel analysis

**DOI:** 10.1038/s41598-020-73572-5

**Published:** 2020-10-02

**Authors:** Bayuh Asmamaw Hailu, Getahun Gebre Bogale, Joseph Beyene

**Affiliations:** 1 Department of Epidemiology and Biostatistics, School of Public Health, College of Medicine and Health Sciences, Wollo University, Dessie, Ethiopia; 2 Department of Health Informatics, School of Public Health, College of Medicine and Health Sciences, Wollo University, Dessie, Ethiopia; 3 Department of Health Research Methods, Evidence, and Impact, McMaster University, Hamilton, Canada

**Keywords:** Health services, Nutrition, Public health, Risk factors

## Abstract

Stunting remains a major public health concern in Ethiopia. Government needs to reshape and redesign new interventions to reduce stunting among under-five children. Hence, this study identified the problem according to location and risk factor. This study is a secondary data analysis of the 2016 Ethiopian Demographic and Health Survey. A total of 9588 children aged 0–59 months were included in the study. The spatial and multilevel logistic regression analyses were used to explore spatial heterogeneity and identify individual- and household-level factors associated with stunting and severe stunting. Spatial heterogeneity of stunting and severe stunting was seen across the study setting. Male children (AOR = 1.51, CI 1.16, 1.96); multiple births (AOR = 27.6, CI 10.73, 71.18); older children (AOR = 1.04, CI 1.01, 1.05) and anemic children (AOR = 3.21, CI 2.3, 4.49) were severely stunted at individual-level factors. Children from educated and malnourished mothers (respectively, AOR = 0.18, CI 0.05, 0.71; AOR = 5.35, CI 3.45, 8.32), and from less wealthier mothers (AOR = 5.95, CI 2.58, 13.69) were severely stunted at household-level factors. Giving priority to the hotspot areas of stunting and older and anemic children, multiple births, and maternal undernutrition is important to reduce stunting. Studies are recommended to fill the gaps of this study.

## Introduction

Stunting is the effect of chronic malnutrition as a result of inability to receive adequate nutrition over a long period and recurrent illnesses. It is a sign of malnutrition and cause of negative health consequences throughout the lifespan, for instance, severe complications at birth, reduced cognitive ability and development, school absenteeism, and poor socio-emotional skills^1–3^. Additionally, chronic diseases are more likely in later lifetime, leading to increased health care costs. Childhood stunting also leads to reduced height in adulthood, which, due to the persistence of shortness over the lifespan, and the negative effect of height on income, also reduces income in adulthood^4^. It is estimated that one in four children under-5 years are failing to grow along the optimum course set out in the WHO’s Child Growth Standards^5^. Global estimates show that one in five children will be stunted in 2020^6^.

Worldwide, stunting is the cause of the death of one million children every year^7^. Though the global burden of stunting reduced between 1990 and 2015 by more than 25%, it has still been a major nutrition-related risk factor causing 257 deaths per 100,000 children^8^. According to the United Nations Children’s Fund (UNICEF)/WHO/World Bank report, 151 million (22%) under five children were stunted at global level. Of this, the low- and lower-middle-income countries contributed to 91% of stunted children, whereas, more than one in three children exists in sub-Saharan Africa countries including Ethiopia^9^. The prevalence of childhood stunting (both stunting and severe stunting) at the population level is as high as 32% in Africa, 24% in Asia and 11% in Latin America^10^.

The reduction of childhood stunting is vital for global health and development^10^. The WHO has set a goal to decrease by 40% the number of stunted children by 2025^4^. But, achieving this goal may face challenges in vulnerable areas such as sub-Saharan Africa, where prevalence of stunting is expected to decrease by only 6% (from 38 to 32%) in this time period, and the number of stunted children is likely to remain unchanged by 2025^10^. The number of stunted children in Africa is projected to increase from 56 million in 2010 to 61 million by the year 2025^9,11^. Ethiopia is one of the countries with the highest number of stunted children at global level^12,13^.

In sub-Saharan Africa, deaths of 136,455 children in 2015 were due to stunting^14^. In Ethiopia, 38% of under-five children were stunted^15^ and it contributed to 960,742 disability adjusted life years (DALYs) and 11,065 deaths in 2015^14^. In general, suboptimal nutrition has been a major causal factor in stunting in low-income countries^16^.

In Ethiopia, about two out of every five children 0–59 months of age are stunted^17^. Though the recent 2016 Ethiopian Demographic and Health Survey (EDHS) report showed that there is a reduction of stunting prevalence among under-five children, its magnitude is still high^17^. Additionally, evidences from different regions of the study setting shown that the prevalence of stunting is to date widely distributed in children of 0–59 months of age with a wide inconsistency through the regions of Ethiopia^17,18^. However, there is a lack of information for severe stunting in the study area as this study is intended to fill this gap.

Worldwide, there are different efforts that have been implemented to combat childhood stunting. Consequently, the United Nations Sustainable Development Goals (SDGs) planned to terminate all kinds of malnourishment by the end of 2030^19^. This plan could be transformed into a 4% annual reduction and then proposed a decreasing in the number of under-five children stunted from 171 million in 2010 to 100 million at the end of 2025. However, at this reduced rate, the magnitude of childhood stunting is predicted to reach not more than 20% (127 million), by 2025 showing that the world will not achieve the plan^20^. As per the report of global progress, the study setting, Ethiopia necessitates a mean of six percent annual decline rate to attain the WHO 2025 plan of 26.8% childhood stunting. However, the present rate of reduction is not more than 2.8%^21^. Thus, Ethiopia is off-track to reach the SDGs of ending child malnutrition by 2030^12^. Therefore, this study is aimed to explore spatial heterogeneity and identify the influencing factors of childhood stunting and severe stunting in Ethiopia by producing the best available evidences for decision-makers to reduce the problem.

## Methods

### Study setting

The study was conducted in Ethiopia, which is located in the North Eastern part of the Africa or known as the “Horn of Africa.” It is bounded by north and south Sudan on the west, Eritrea and Djibouti to the northeast, Somalia to the east and southeast, and Kenya to the south. Ethiopia lies between the 3° N and 15° N Latitude and 33° E and 48° E Longitude^21,22^.

The country occupies an area of approximately about 1,127,000 km^2^, which is almost twice the size of France. The Ethiopian landmass consists of a large, high elevated plateau bisected by the Rift Valley into the northwestern and the southeastern highlands, each with associated lowlands. The contrast in relief is remarkable as land elevation ranges between − 155 m of Asal Lake in the Afar depression (the lowest point in Africa) to the peak of Mt. Ras-Dejen, at 4620 m above sea level in the Semen Mountains^23^. There are nine regional states and two city administrations subdivided into 68 zones in the administrative structure of the country^24^.

### Study design

Population-based cross-sectional study was employed to explore geographical variation and identify the influencing factors of childhood stunting and severe stunting in Ethiopia.

### Data source

The data for this study were taken from the 2016 EDHS. The 2016 EDHS is the fourth comprehensive and nationally representative survey conducted in Ethiopia as part of worldwide Demographic and Health Surveys (DHS) project. The main objective of the 2016 EDHS was to provide timely and reliable data on health and demographic outcomes at both national and regional levels^25^. The EDHS 2016 data were downloaded from The DHS website after being granted permission. More detailed information on DHS survey design and nutrition data has been summarized^25^. The 2016 EDHS database used for this analysis was prepared in seven data structures, namely, Household (HR), People-all household members (PR), Women with complete interviews (IR), Men with complete interviews (MR), Couples (CR), Children < 5 of interviewed women (KR), and All births to interviewed women (BR) data files. Among them, the KR’s data file (dataset) was used for this analysis.

### Measurement of variables

Children’s height was measured preceding the survey in all of the selected households. Each team of data collectors carried a weighing scale and measuring board. Measurements were made using digital screens, lightweight SECA scales designed and manufactured under the authority of the UNICEF. The measuring boards employed were specially made by Shorr Productions for use in survey settings. Children under the age of 2 were measured lying down on the board (recumbent length), and older children were measured on standing height^17^.

The height-for-age index of children was calculated using growth standards published by the WHO. These growth standards were generated through data collected in the WHO Multicenter Growth Reference Study^26^ and expressed in standard deviation units from the Multicenter Growth Reference Study median. The height-for-age index is an indicator of linear growth retardation and cumulative growth deficits in children^27^. Children with height-for-age Z-score below minus two standard deviations (− 2 SD) from the median of the WHO reference population^28^ are considered to be stunted (moderate and severely stunted) while children who are below minus three standard deviations (− 3 SD) from the reference median are considered severely stunted^17,29^.

Blood specimens for anemia testing were collected from all children ages 6–59 months from a heel prick in the case of children age 6–11 months and collected in a microcuvette. Hemoglobin analysis was carried out on-site using a battery-operated portable HemoCue analyzer. As a result, child’s anemia status was classified as, based on hemoglobin level, anemic (< 11.0 g/dl), mildly anemic (10.0–10.9 g/dl), moderately anemic (7.0–9.9 g/dl), severely anemic (< 7.0 g/dl), and not anemic (≥ 11.0 g/dl)^17^.

### Sample size and sampling procedures

Every 5 years, the EDHS has collected data on nationally representative samples of key indicators including childhood stunting. A stratified two-stage cluster sampling was employed where the enumeration areas (EA) were primary sampling units and households were secondary sampling units. An EA is a geographic area covering on average 181 households. Data was collected by trained interviewers from 645 EAs (202 urban and 443 rural areas) using systematic random sampling proportional to size. A fixed number of 28 households per enumeration area were selected for households’ sample frame. A structured and pretested questionnaire including < 5 children characteristics used for the study. After cleaning of the under-five children’s dataset which does not have location data, the total sample of 9588 individuals from 6406 households, out of which 4695 females and 4893 males were included in the study. All sampling procedures, data collection and data quality control were done by a DHS team^17^.

### Data processing and analysis

#### Spatial autocorrelation (Global Moran’s I) analysis

Statistically significant clusters are defined as geographic areas in which the magnitude of stunting and severe stunting is disproportionately higher compared to neighboring areas. Global Moran’s *I* tests detect the existence of at least one cluster with the specific location of the study setting^30^. To detect the presence of clustering in the study setting, Global Moran’s *I* test was performed in Geographic Information System (GIS). Global Moran’s *I* statistics were calculated, a value close to − 1 indicates dispersion and Moran’s *I* value close to + 1 indicates clustering, whereas Moran’s *I* value of zero indicates the random distribution^31^. In this study, spatial analysis was performed using the spatial statistics tools in GIS (Getis-OrdGi* statistics, Anselin Local Moran’s *I*) which have been widely used to detect clusters in a variety of health-related fields^29^.

#### Cluster-outlier (Anselin Local Moran’s I) analysis

Local Moran index, also called Local Indicator of Spatial Association (LISA), was used to identify local clusters. Four types of spatial autocorrelations were observed: high–high (high value of cases surrounded by high values), low–low (a low value of cases surrounded by low values), and high–low or low–high (a high value of cases surrounded by low values or vice versa)^32^. Association between areas of similar values (such as high–high and low–low) were defined as positive spatial autocorrelation, whereas the association between areas of dissimilar values (low–high and high–low) were considered negative autocorrelation (outliers)^24,30,31^. In this study, spatial heterogeneity refers to the uneven distribution of stunting and severe stunting across the regions of Ethiopia.

### Multilevel logistic regression

Multilevel logistic regression was utilized to assess the impact of measured individual and household level factors. Multilevel analysis was considered appropriate in order to account for the hierarchical nature of the EDHS data and to be able to estimate household level effects on the outcome variable^33–35^. The multilevel logistic regression model was applied in the study and this consists of two sub models at level 1 and level 2. This implies that individuals (level 1) were nested within the household (level 2). The level 1 model represents the relationships among the individual level variables, while the level 2 model examines the influence of household level factors.

### Model building

In the above model selection, mixed-effect logistic regression was selected as the best model. Afterwards, the next four models building and analysis were done using STATA 14. Overall, four models containing variables of interest were fitted for each of the under-5 stunted children. The first model (M_0_) is an empty model which was fitted without independent variables to test random variability using Intra House Hold Correlation (IHHC). IHHC measures the proportion between households. The IHHC was calculated to evaluate whether the variation in stunting is primarily within or between households. IHHC refers to the ratio of the between-household variance of the total variance and it tells us the proportion of the total variance in the outcome variable that is accounted at household level and its value less than 5% were considered as the level of significance^36^. The second model (M_1_) was fitted to all lower-level (individual level) factors, the third model (M_2_) used for all higher-level (household level) factors; and the fourth model (M_3_) used for both lower- and higher-level factors to report. The proportional change in variance (PCV) measures the total variation attributed by individual level factors in the multi-level model. Model fitness for the report selected by using AIC and BIC, which indicate the smallest, is the best for final report.

### Ethics declarations

Ethical clearance with written consent (Authorization Letter_121143) was obtained from Measure DHS International Program which authorized the data sets. All the data which used in this study are publicly available, aggregated secondary data with not having any personal identifying information that can be linked to particular individuals, communities, or study participants. Confidentiality of data maintained anonymously.

## Results

### Spatial variation of childhood stunting and severe stunting

**Figure 1:**
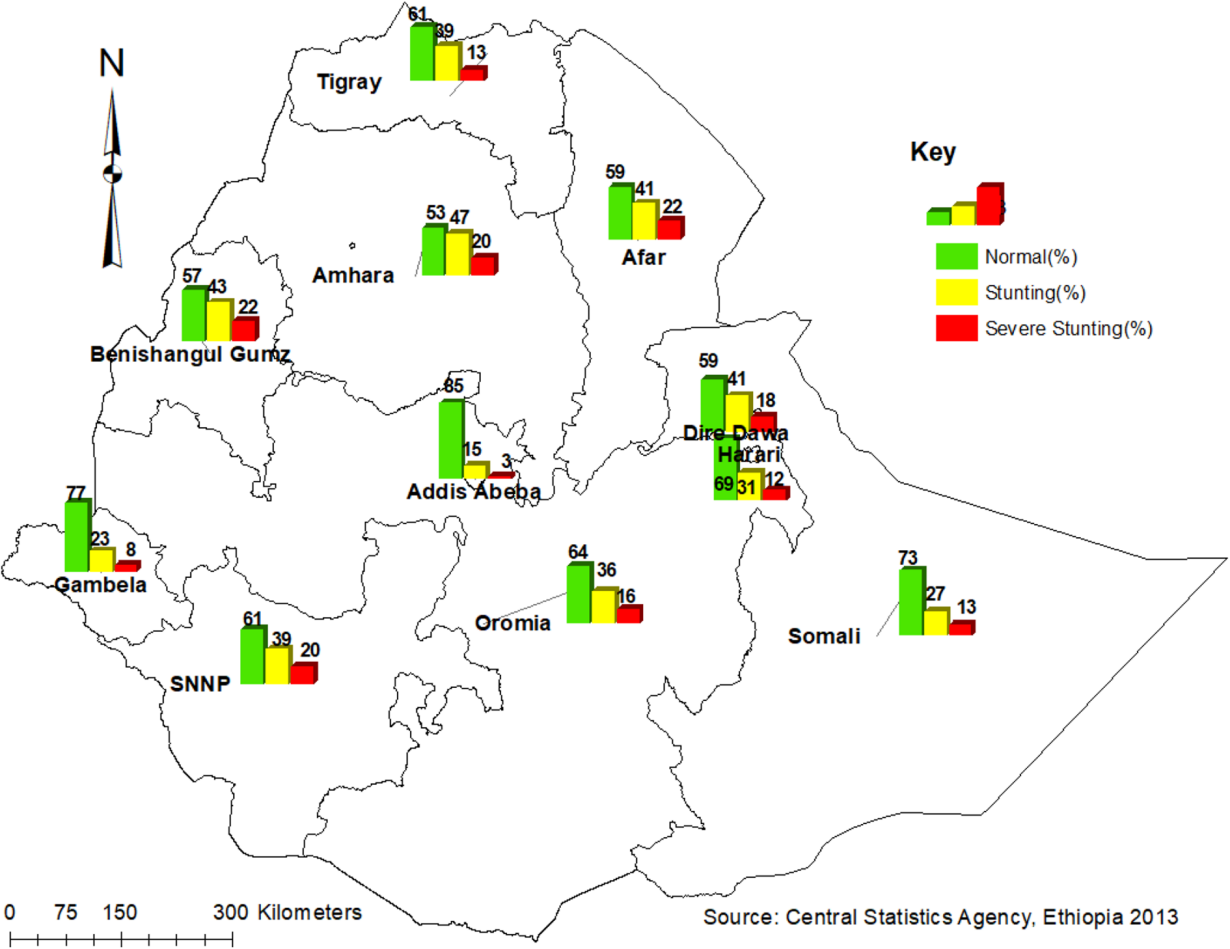
Weighted prevalence of stunting and severe stunting among children by regions in Ethiopia, 2016. The map was created by the author(s) using ArcGIS version 10.3 (URL: https://www.esri.com/software/arcgis). The red color coded bars indicate the prevalence of severe stunting (< − 3 SD), the yellow color coded bars indicate the prevalence of stunting (< − 2 SD), while the green color coded bars indicate the non-stunted children.

The five highest regions with childhood stunting were Amhara (47.2%), Benishangul Gumuz (42.8%), Afar (40.7%), South Nations Nationality People (SNNP) (39.1%) and the Tigray (38.9%). The four highest severe childhood stunting regions were Benishangul Gumuz and Afar (22%), and SNNP and Amhara (20%). In Fig. 1, the bar chart of the red color indicates severe stunting, the yellow color for stunting, and the green color for not stunted Fig. 1.

### Spatial autocorrelation (Global Moran’s I) analysis

**Figure 2:**
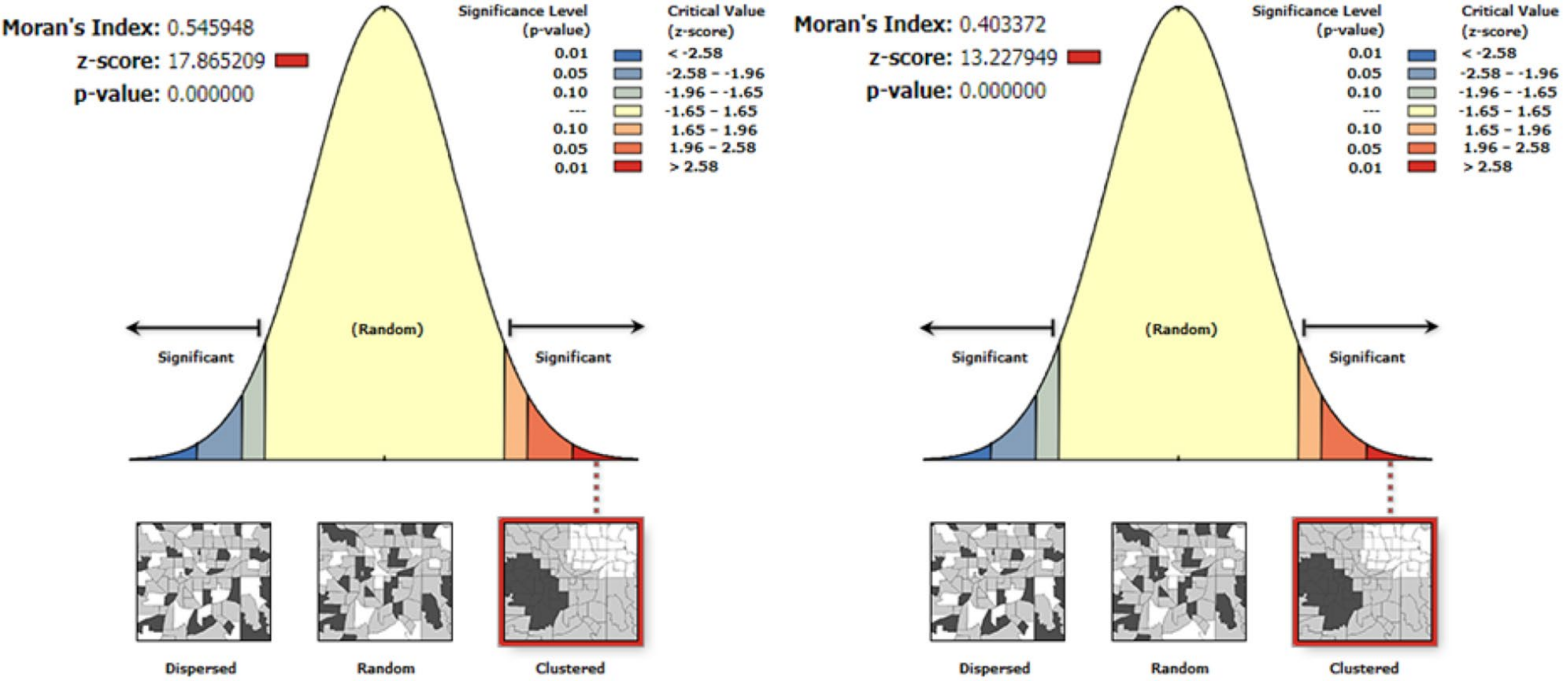
The spatial pattern of childhood stunting and severe stunting in Ethiopia 2016. The map was created by the author(s) using ArcGIS version 10.3 (URL: https://www.esri.com/software/arcgis). The right side of each panel shows that high rate of stunting occurred over the study area. The output includes automatically generated keys on the right and left side of e of each panel. The auto-generated interpretations displayed underneath each panel show that the likelihood of clustered pattern occurring by chance is less than 1%. The bright red and blue colors (in the tails) indicate the increased significance level. Z-scores reflect the intensity of spatial clustering, and statistically significant peak Z-scores (deep sky-blue color) indicate distances where spatial clustering is pronounced. The dark red color indicates significant global clusters.

The spatial patterns of stunting and severe stunting were found to be non-random. The Global Moran’s *I* values were 0.55 and 0.40 for stunting and severe stunting, respectively. These values indicate significant clustering of stunting and severe stunting across the country. The clusters’ patterns were > 99% significant at *p* < 0.001 Fig. 2.

The spatial autocorrelation analysis showed statistically significant variations, because the observed values in the Moran’s *I* indexes are greater than the expected values, and the *p* values are less than 0.001 (statistically significant at 99%) Table1.

**Table 1:** Spatial autocorrelation analysis of childhood stunting and severe stunting in Ethiopia, 2016.

Type of stunting	Observed Moran's *I* index	Expected Moran's *I* index	*Z*-score	*P* value
Stunting	0.545948	− 0.001613	17.865209	< 0.001
Severe stunting	0.403372	− 0.0011613	13.227949	< 0.001

### Cluster outlier (Anselin Local Moran’s I) analysis

**Figure 3:**
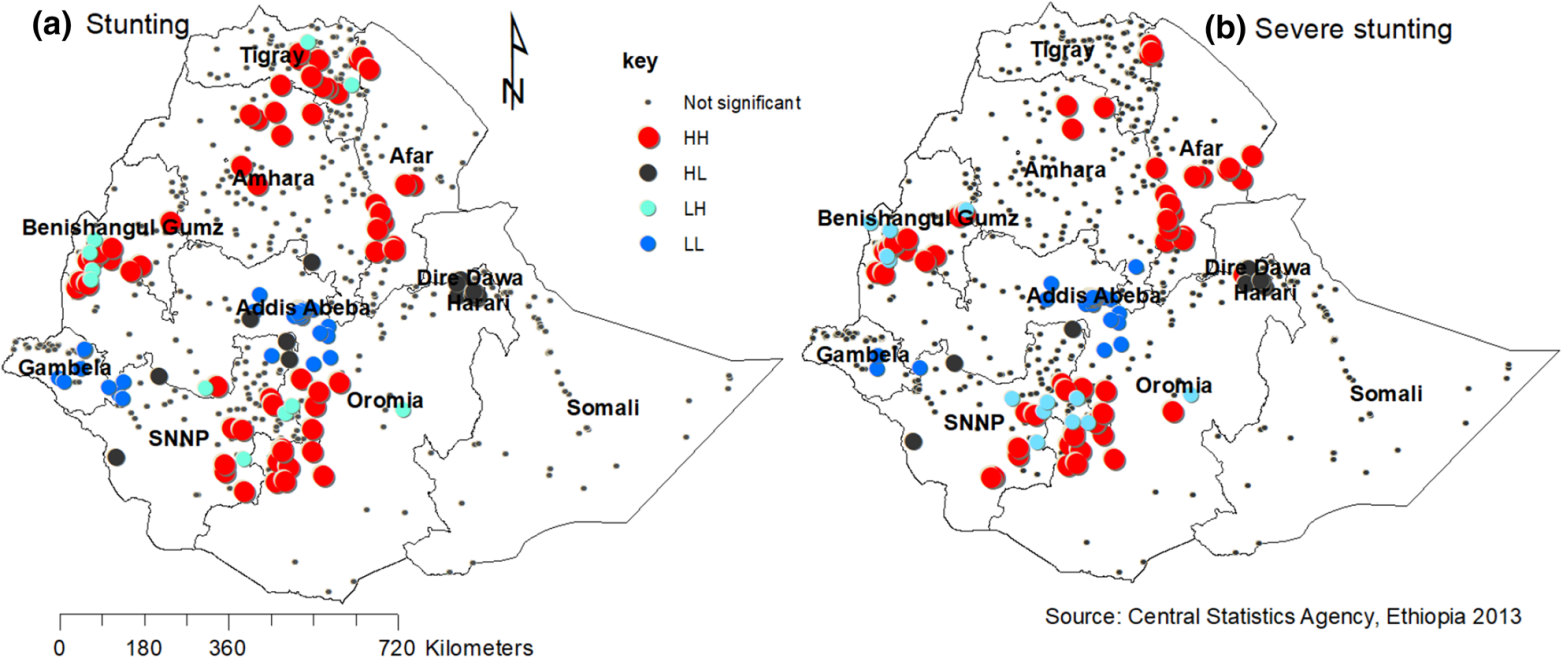
Cluster outlier identification of childhood stunting and severe stunting with its severity in Ethiopia, 2016. The map was created by the author(s) using ArcGIS version 10.3 (URL: https://www.esri.com/software/arcgis). Each point data on the map represents a single enumeration area with a number of stunted and severely stunted children. HH (high–high) means high rates of stunted and severely stunted children surrounded by similar characteristics; HL (high–low) means high rates of stunted and severely stunted children surrounded by low rates of stunted and severely stunted children; LH (low–high) means low rates of stunted and severely stunted children surrounded by high rates of stunted and severely stunted children; and LL (low–low) means low rates of stunted and severely stunted children surrounded similar characteristics. The red color (HH) indicates hotspot areas of stunting and severe stunting; the blue color (LL) indicates cold-spot areas of stunting and severe stunting; and the black (HL) and aqua (LH) colors indicate outliers. The hotspots are public health important.

Figure 3 shows the geographical distribution of stunting and severe stunting in the country with specific clusters and the characteristics of their surrounded areas. In both panels the red color indicates a high rate of stunting surrounded by high rates of similar cases. Such specific areas are in Amhara (eastern north Gondar and southwest south Gondar zones), Benishangul Gumuz (part of Assosa and Kemash); Afar (Zone 2 at the boundary of Tigray, Zone 1 and 3 at the boundary of Amhara); SNNP (north Omo, Gedio and Sedamo zones in the border area of Oromia); Oromia (west Welega, Jima and Borena at the border of SNNP, and Arisi zones); and Tigray (central, eastern and southern parts) regions are stunted areas of the country and surrounded by similar characteristics Fig. 3a.

Likewise, the Amhara (north and south Gondar, and Waghmira zones); Benishangul Gumuz (Assosa areas, some part of Metekel and Kemash); Afar (Zone 2 at the boundary of Tigray, Zone 3 at the boundary of Amhara and some part of Zone 1); Oromia (Borena at the boundary of SNNP); and SNNP (some parts of south and north Omo zones) regions and Dire Dawa city administration are severely stunted areas surrounded by similar characteristics Fig. 3b.

### Model comparison

In this study IHHC value was 17.1% and 15.35% for stunted and severely stunted respectively, which is > 5%, it indicates the level analysis is more appropriate than a binary logistic regression model. AIC and BIC measure, model fitness for report, which indicates the smallest (M_3_) was the best model for the final report Table 2.

**Table 2:** Model comparison for report among M_0,_ M_1,_ M_2_ and M_3_.

Random effect	Empty (M_0_)		Individual (M_1_)		Household (M_2_)		All (M_3_)	
	Stunted	Severely stunted	Stunted	Severely stunted	Stunted	Severely stunted	Stunted	Severely stunted
Variance	19.2	11.64	18.1	11.44	19.1	11.51	18.02	11.29
IHHC (%)	17.1^a^	15.35 ^a^	21.7	19.78	13.8	12.99	19.4	18.68
PCV	Reference	Reference	5.73	1.72	0.52	1.12	6.15	3.01
Log likelihood	− 6052	− 3754	− 5152	− 3343	− 5824	− 3578	− 5005	− 3239
AIC	12,110	7514	10,324	6706	11,678	7186	10053^b^	6522^b^
BIC	12,131	7535	10,394	6775	11,784	7292	10205^b^	6674^b^

^a^Variability between household level.

^b^Selected models for report.

### Individual and household level characteristics of children aged 0–59 months

A total sample of 9588 children aged 0–59 months, was included in the study. The prevalence of stunting was 38.4% (CI 37.4%, 39.4%) and severe stunting was 17.4% (CI 16.7%, 18.2%) among under-5 children across the country. Children’s mean (± SD) age was 28.64 (± 17.56) months, maternal mean age was 29.5 (± 6.6) years and the mean household size was 6.1 (± 2.1). Out of the total sample, 51% were males, 2.4% twins, 32% above moderate anemic, 65.6% of children born from uneducated mother, 18% of the children’s mothers were stunted and wasted, 46.5% of children from households with poor wealth index, 65.6% of children of mothers with no education, and 86.6% were male from household heads Supplementary Table S1.

### Multilevel regression for stunting among children aged 0–59 months

After controlling for other individual and household level factors; at lower (individual/child) level characteristics such as the child’s age, sex, multiple birth, child fever and anemia; and at higher (household) level characteristics such as maternal education, maternal stunting and wasting and wealth index were statistically significant factors for stunting among children.

When a child age increased by one month, the risk of stunting was increased by 4%. Male children were 89% more likely to be stunted than females. Children with multiple (twin) births had 25 times higher risk of stunting as compared to those with single births. Children with fever were 66% higher odds of stunting as compared to the normal. Children with mild, moderate, and severe anemia were 2.2, 3.18 and 5.49 times more likely to be stunted, respectively, than those of not anemic Supplementary Table S1.

Children born from non-educated mothers were 59% more likely to be protected from stunting than those born from educated mothers. Children from stunted mothers were 6.94 times more exposed to stunting as compared to those from normal. Children from wasted mothers were 76% more likely to be exposed to stunting. Children whose household wealth index was rich, middle, poorer and poorest had 2.24, 3.21, 5.75 and 4.48 times higher risk of stunting, respectively, as compared to children from richest household Supplementary Table S1.

### Multilevel regression for severe stunting among children aged 0–59 months

All variables associated with childhood stunting; except child fever and maternal wasting; were also associated to severe stunting among children aged 0–59 months. When a child age increased by one month, the risk of severe stunting was increased by 4%. Male children were 51% more likely to be severely stunted than females. Children with multiple (twin) births had 28 times more likely to be severely stunted as compared to those with single births. Children with mild, moderate, and severe anemia were 3.21, 3.55 and 10.79 times more likely to be severely stunted, respectively, than those of not anemic Supplementary Table S1.

Children born from non-educated mothers were 82% more likely to be protected from severe stunting than those born from educated mothers. Children from stunted mothers had 5.4 times more likely to be exposed to severe stunting as compared to those from normal. Children whose household wealth index were middle, poorer and poorest had 3.25, 6.19 and 5.95 times higher risk of severe stunting, respectively, as compared to children from the richest households Supplementary Table S1.

## Discussion

This study indicated that childhood stunting and severe stunting at national and regional levels are non-random. Significant clusters of stunting were detected in Amhara, Benshangul Gumuz, Afar, SNNP, and Tigray regions. Based on the United Nations Children’s Fund stunting framework^37^, both individual and household level factors were identified. Thus, child age and sex, multiple birth, child’s fever and anemia, poor household wealth index, maternal education, and maternal stunting and wasting were statistically significant factors associated with stunting and severe stunting among children 0–59 months in Ethiopia.

The spatial variation of childhood stunting and severe stunting Fig. 1 showed the high burden regions (Amhara, Benishangul Gumuz, Afar, SNNP, and Tigray). It may be due to less economic status of the community in the regions. The spatial autocorrelation analysis Fig. 2, Table 1 revealed that childhood stunting and severe stunting had a high spatial dependency (respectively, Moran’s I: 0.55 and 0.40) in agreement with studies done in India^38,39^. The LISA results Fig. 3 showed heterogeneity of childhood stunting clustering in Amhara, Benishangul Gumuz, Afar, SNNP, Oromia, and Tigray regions. Similarly, with the exception of Tigray region, all regions, including rural areas of Dire Dawa city administration are hotspot areas of severe stunting. It is in line with a study done in the same setting^40^. The possible explanation of the specific hotspot areas (administrative zones) may be related to poor feeding practices and repeated drought. Such hotspot areas may need governmental and partners attentions towards nutritional interventions. Socio-cultural feeding practices shall be studied to identify causes of high rates of stunting.

According to this finding, an increase in child age contributed to the increasing risk of stunting and severe stunting in under-five children. This result is in line with other studies^40–42^. It may be due to the fact that poor complementary feeding practices (or the complimentary foods use directly in the regular household-diet which is made of cereal or starchy root crops during this age period) among older children in the study area which may lead to growth retardation and both types of stunting. However, it is contradicted by other studies done in Indonesia^43^ and Bhutan^44^. The difference might be due to cultural variations in child feeding practices across the study settings. Nutritional education shall be given to the community against the poor feeding practices for older under-five children.

Male children were more stunted than females. The result is agrees with other findings done in Ethiopia and Nigeria^27,41,45,46^. Evidence of a higher risk in males under the age of five would be faster growth (hence higher dietary requirements) in this year of life and the other reasons may be hormonal and genetic factors. Multiple births were strongly associated with childhood stunting and severe stunting. This finding is similar to another study done in Cambodia^47^. This could be attributed to inadequate breastfeeding, low birth weight and competition for nutritional intake, which happen more in children of multiple births.

The children with anemia were more exposed to both stunting and severe stunting. This finding is in line with other findings^48,49^. In fact, anemia is not directly affecting stunting but may be an indirect association with shorter night sleeping duration and higher frequency of night waking. Infants with anemia and stunting are thought to be less proficient at regulating their emotions^50^, and to explore and interact less with their environment than well-nourished children. The other possible explanation may be that anemia has possible relationships with intestinal parasitic infections, so children with this situation may be more affected by stunting^51,52^. Since older children are more exposed to contaminations which may cause parasitic infections, nutritional counseling shall be given to mothers during their postnatal service. This study demonstrated that under-five children with fever in the last two weeks of the survey had a positive association with stunting, which is supported by studies from Ethiopia^45,46^. In fact, there are underlying conditions (including fever) that facilitate childhood stunting.

Surprisingly, in this finding children of more educated mothers were more likely exposed to stunting and severe stunting compared to those whose mother completely uneducated. The reason might be that educated mothers have their own works and left their children to other third parties (care givers) and the children can get inadequate nutrition with lack of breastfeeding. Moreover, it might be due to poor mother–child interaction between child and educated mother, that might trigger the occurrence of childhood stunting and severe stunting^53,54^. Mass media promotion may help the care givers to have better awareness towards child feeding practices. On the other hand, the main work of uneducated mothers is caring and educating their children (including breast feeding in addition to hygiene and different personal cares) in close proximity, that might cause for reduction in stunting and severe stunting. In fact, this finding is in contrary with many other scholars. The advantage of improved maternal education helps the mother’s ability to make better decisions for herself and her children and can be linked to the ability of mothers to make healthier choices in caring practices. Likewise educated mothers better equipped to offer their children good care. In general different scholars assure that maternal education protects childhood stunting^11,41,45,47,55^.

This study showed when the household income increases the burden of stunting and severe stunting in children decreases. This finding is in line with other findings done in Bhutan, Nigeria and Congo^27,44,56^. This finding suggests that the children’s stunting status depends upon the socioeconomic status of their household. It is linked to a poor child feeding practices that contribute to stunting and severe stunting include suboptimal breastfeeding (specifically, non-exclusive breastfeeding) and inadequate quantity, limited quality and variety of complementary feedings. As a result of poor socioeconomic status of a household, inadequate food security pushes the caregiver to negligence, non-responsive feeding practices, and inadequate child stimulation can all interrelate to inhibit the growth and development of the child.

Stunting represents the chronic state of under nutrition^17^ that often begins in the uterus due to maternal under nutrition^57^. In this finding maternal stunting and wasting were inversely associated with children linear growth or directly associated with stunting, however, maternal stunting was allied to severe stunting only, which is similar with other findings^58,59^. The Federal Ministry of Health may need to prepare nutritional counseling packages and implement at the health care facilities during antenatal and postnatal services.

In general, the government needs to implement the recent National Food and Nutrition Policy, different Global and National Initiatives like Sustainable Development Goals, Seqota Declaration, and Agriculture Extension Program Development Army (Women and Health Development Armies). That means it needs intersectoral collaborations with strong political commitment at the national level to improve public health infrastructure in both rural and urban areas; and improve global and local partners and civil society organizations working on nutrition major funding opportunities, initiatives and strong public–private partnership at the national and global level.

Even though this study was based on national representative data and used a combination of testing methods, it has its own limitations. One limitation is that the cross-sectional nature of the study that causes the results cannot be used to establish cause and effect relationship. The DHS Global Positioning System (GPS) data cannot indicate the exact location of study participants, since it was collected in enumeration area and random shifting of location data made for both urban and rural settings. Another limitation is the absence of other important variables like cultural and behavioral factors to validate this finding.

## Conclusions

This Geo-spatial analysis showed that stunting and severe stunting among childhoods were non-random in Ethiopia. This finding explored higher hot spot areas of regions such as Amhara, Afar, Benishangul Gumuz, Oromia, and SNNP, which need an intervention. Whereas, Somali and Gambela regions are cold spot areas of stunting. Therefore, it may need further detailed study why happens this wide gap between the regions. Statistically significant factors associated with stunting and severe stunting among children 0–59 months in Ethiopia were age of children (younger), multiple birth, sex of children (males), child’s fever and anemia, poor household wealth index, higher maternal education, and maternal stunting and wasting.

To decrease the hot spots of stunting and severe stunting among Ethiopian children, giving priority attention to household wealth improvements may be important. Household counseling for caregivers, which should be aimed at improving the nutritional status of childhoods, hygienic practices against parasitic infections, and underlying conditions is also recommended in order to achieve optimal brain development and reduce mortality triggered by malnutrition especially severe stunting. Reaching the SDGs nutrition and food security target will require a systematic effort to maximize the efficiency and impact of nutrition budgets. It is recommended that Optima Nutrition can be a useful in informing priority setting and allocation of resources across multi-factorial nutritional interventions.

## Supplementary information

Supplementary Table S1.

## Data Availability

The datasets used for this study, the ‘2016 Ethiopian Demographic and Health Survey’, were obtained from The DHS program (www.dhsprogram.com), but the ‘Dataset Terms of Use’ do not permit us to distribute this data as per data access instructions (https://dhsprogram.com/data/Access-Instructions.cfm). To get access for the dataset anyone must first be a registered user of the website (www.dhsprogram.com), and download the 2016 Ethiopian Demographic and Health Survey (Survey and GPS datasets).

## Abbreviations

AOR: Adjusted odds ratio
CI: Confidence interval
DHS: Demographic and health survey
EDHS: Ethiopia demographic and health survey
HAZ: Height-for-age Z-score
IHHC: Intra house hold correlation
SD: Standard deviation
SDGs: Sustainable development goals
SNNP: South Nations Nationality People
UNICEF: United Nations children’s fund
WHO: World Health Organization
