# Supplementary material for: Spatial heterogeneity and factors influencing stunting and severe stunting among under-5 children in Ethiopia: spatial and multilevel analysis
Source: Sci Rep. 2020 Oct 2;10:16427. doi: 10.1038/s41598-020-73572-5 (PMC7532151; doi:10.1038/s41598-020-73572-5)
Supplement: Supplementary file 1 — Supplementary Table S1. [file 41598_2020_73572_MOESM1_ESM.pdf]

Spatial heterogeneity and factors influencing stunting and severe stunting among under-5 children in Ethiopia: Spatial and Multilevel Analysis

Authors

1. Bayuh Asmamaw Hailu
2. Getahun Gebre Bogale
3. Joseph Beyene

Supplementary Table S1. Individual and household level factors and multilevel logistic regression analysis for stunting and severe stunting of under-5 children in Ethiopia, 2016.

Characteristics	Weighted sample size (%)	Weighted Prevalence(No)		Odds Ratio(95%CI)			
		Stunted	Severely stunted	Crude		Adjusted	
				Stunted	Severely Stunted	Stunted	Severely Stunted
**All**	9588(100)	38.4(3681)	17.4(1669)				
**Individual(Lower Level) Characteristics**							
Child age	9588(100)	38.4(3681)	17.4(1669)	1.05(1.04,1.05)	1.05(1.04,1.05)	1.04(1.03,1.05)**	1.04(1.01,1.05)**
**Type of birth**							
Single	9363(97.6)	38(3556)	17(1601)	1	1	1	1
Twin	225(2.4)	55.5(125)	30(68)	13.8(7,27)	10.5(4.9,22.7)	25.2(11.1,57.5)**	27.6(10.73,71.18)**
**Child sex**							
Female	4695(49)	35.4(1664)	16(739)	1	1	1	1
Male	4893(51)	41(2017)	19(929)	1.81(1.52,2.16)	1.48(1.2,1.8)	1.89(1.53,2.33)**	1.51(1.16,1.96)**
**Place of delivery**							
Health center	2573(26.8)	30.5(786)	11(290)	1	1	1	1
Not health center	7015(73.2)	41.3(2895)	20(1355)	2.6(2.08,3.25)	3.6(2.7,4.9)	0.91(0.68,1.23)	1.39(0.95,2.03)
**Have diarrhea**							
No	8419(88)	38.1(3209)	17(1454)	1	1		
Yes	1151(12)	40.8(469)	19(215)	1.17(0.88,1.56)	1.18(0.84,1.65)		
**Have fever**							
No	8167(85.3)	37.9(3096)	17(1427)	1	1	1	
Yes	1411(14.7)	41.5(585)	17(242)	1.32(1.02,1.71)	0.91(0.7,1.2)	1.66(1.21,2.26)**	
**Anemia status**							
Not anemic	3565(43)	37(1330)	15(542)	1	1	1	1
Mild	2090(25)	44(917)	20(427)	1.7(1.4,2.2)	2.2(1.6,2.79)	2.2(1.68,2.88)**	3.21(2.3,4.49)**
Moderate	2439(29)	44(1074)	22(535)	2.1(1.6,2.7)	2.1(1.6,2.8)	3.18(2.42,4.18)**	3.55(2.54,4.96)**
Severe	252(3)	53(133)	31(78)	3.8(2.1,6.62)	6.7(3.4,13.1)	5.49(3.01,10.01)**	10.79(5.23,22.26)**

**Household (Higher Level) Characteristics**							
**Mother age**	9588(100)	38.4(3681)	17.4(1669)	1.03(1.01,1.04)	1.02(0.99,1.04)	0.99(0.97,1.02)	
**Maternal occupation**							
No	6994(73)	38.3(2675)	18(1224)	1	1		
Yes	2594(27)	38.8(1006)	17(445)	1.05(0.81,1.36)	0.9(0.7,1.2)		
**Mother Marital status**							
Married	9011(94)	38.3(3447)	17(1569)	1	1		
Not married	577(6)	40.5(234)	17(100)	1.08(0.68,1.72)	0.7(0.4,1.2)		
**Mother education**							
Secondary and above	679(7.1)	20.1(137)	6(39)	1	1	1	1
Primary	2615(27.3)	35.3(924)	15(388)	4.94(3.09,7.91)	8(3,21)	0.77(0.56,1.08)	0.85(0.57,1.25)
No education	6294(65.6)	41.6(620)	20(1669)	10.35(6.61,16.23)	18.4(7,47)	0.41(0.21,0.81)**	0.18(0.05,0.71)**
**Mother's stunting status**							
Normal	7765(82)	35(2738)	15(1202)	1	1	1	1
Stunted	1443(15)	52(746)	26(378)	5(3.7,7.2)	4(2.8,5.9)	6.94(4.64,10.36)**	5.35(3.45,8.32)**
Severely stunted	269(3)	57(154)	27(73)	8(4.1,17.1)	5.8(2.6,13)	12.11(5.1,28.71)**	3.15(0.77,12.83)
**Mother's wasting status**							
Normal	7796(82)	37(2870)	16(1287)	1	1	1	1
Wasted	1571(17)	45(712)	22(342)	2.4(1.8,3.3)	2.3(1.6,3.2)	1.76(1.07,2.88)**	1.7(0.96,2.99)
Severely wasted	91(1)	43(39)	24(22)	1.9(0.69,5.4)	4(1.3,13)	1.35(0.39,4.67)	3.15(0.77,12.83)
**Mother's Body Mass Index**							
Not under weight	7627(80)	38(2878)	17(1301)	1	1	1	1
Underweight	1866(20)	41(767)	19(354)	1.6(1.2,2.1)	1.5(1.1,2.10)	0.91(0.57,1.45)	0.84(0.48,1.47)
**Household wealth index**							
Richest	1367(14.3)	25.5(349)	10(132)	1	1	1	1
Richer	1742(18.2)	34.7(605)	14(244)	3.09(2.06,4.62)	3.3(1.8,5.9)	2.24(1.21,4.13)**	2.02(0.85,4.77)
Middle	2018(21)	37.7(761)	16(320)	4.26(2.87,6.33)	4.4(2.5,7.7)	3.21(1.74,5.93)**	3.25(1.39,7.62)**

Poorer	2249(23.5)	43.1(969)	20(461)	7.17(4.83,10.65)	8.7(5,15)	5.75(3.1,10.67)**	6.19(2.67,14.35)**
Poorest	2211(23)	45.1(997)	23(506)	6.62(4.53,9.67)	10.4(6.1,17.8)	4.48(2.43,8.24)**	5.95(2.58,13.69)**
**Sex of household head**							
Male	8304(86.6)	38.2(3174)	17(1425)	1	1		
Female	1284(13.4)	39.5(507)	19(244)	0.9(0.65,1.23)	0.96(0.7,1.4)		
**Residence**							
Urban	1048(11)	26.2(274)	11(115)	1	1	1	1
Rural	8540(89)	39.9(3407)	18(1554)	4.78(3.37,6.77)	5.4(3.2,8.9)	1.5(0.82,2.74)	1.37(0.58,3.22)
**Household Size**	9588(100)	38.4(3681)	17.4(1669)	1.05(0.99,1.11)	1.05(0.99,1.13)		

Note: ** statistically significant at p<0.05
